# Interferon γ and α Have Differential Effects on SAMHD1, a Potent Antiviral Protein, in Feline Lymphocytes

**DOI:** 10.3390/v11100921

**Published:** 2019-10-09

**Authors:** Peyman Asadian, Dorothee Bienzle

**Affiliations:** Department of Pathobiology, University of Guelph, Guelph, ON N1G 2W1, Canada; pasadian@uoguelph.ca

**Keywords:** interferon, interleukin, IL2, phosphorylation, pSAMHD1, restriction factor, triphosphohydrolase

## Abstract

Sterile alpha motif and histidine/aspartic domain-containing protein 1 (SAMHD1) is a protein with anti-viral, anti-neoplastic, and anti-inflammatory properties. By degrading cellular dNTPs to constituent deoxynucleoside and free triphosphate, SAMHD1 limits viral DNA synthesis and prevents replication of HIV-1 and some DNA viruses such as HBV, vaccinia, and HSV-1. Recent findings suggest SAMHD1 is broadly active against retroviruses in addition to HIV-1, such as HIV-2, FIV, BIV, and EIAV. Interferons are cytokines produced by lymphocytes and other cells that induce a wide array of antiviral proteins, including some with activity again lentiviruses. Here we evaluated the role of IFNs on SAMHD1 gene expression, transcription, and post-translational modification in a feline CD4^+^ T cell line (FeTJ) and in primary feline CD4^+^ T lymphocytes. SAMHD1 mRNA in FetJ cells increased in a dose-related manner in response to IFNγ treatment concurrent with increased nuclear localization and phosphorylation. IFNα treatment induced SAMHD1 mRNA but did not significantly alter SAMHD1 protein detection, phosphorylation, or nuclear translocation. In purified primary feline CD4^+^ lymphocytes, IL2 supplementation increased SAMHD1 expression, but the addition of IFNγ did not further alter SAMHD1 protein expression or nuclear localization. Thus, the effect of IFNγ on SAMHD1 expression is cell-type dependent, with increased translocation to the nucleus and phosphorylation in FeTJ but not primary CD4^+^ lymphocytes. These findings imply that while SAMH1 is inducible by IFNγ, overall activity is cell type and compartment specific, which is likely relevant to the establishment of lentiviral reservoirs in quiescent lymphocyte populations.

## 1. Introduction

Maintaining deoxynucleoside triphosphate (dNTP) concentration within an optimal range is critical for genomic stability and cellular metabolism [1]. The concentration of cellular dNTPs depends on a balance between synthesis, degradation by catabolic enzymes, and use by DNA polymerases [2,3]. Sterile alpha motif and histidine-aspartic domain (HD) containing protein 1 (SAMHD1) is a member of the enzymatic network that regulates the size of the dNTP pool [4,5]. SAMHD1 is biologically active in its tetrameric form and degrades dNTPs to their constituent deoxynucleosides and free triphosphates [4,6,7]. SAMHD1 is also known as a cellular restriction factor that, at least in part, inhibits replication of HIV-1 in myeloid and resting T cells [8,9,10]. The precise mechanism by which SAMHD1 prevents viral replication remains to be determined, but findings to date suggest that SAMHD1 reduces the intracellular dNTP concentration below the threshold required for synthesis of viral genomic DNA, which prevents viral replication [4,11]. Viral cDNA synthesis is an essential early step in the retrovirus life cycle. By limiting DNA synthesis, SAMHD1 decreases replication of retroviruses such as HIV-1, and DNA viruses such as vaccinia and herpes simplex virus type 1 (HSV-1) in dendritic and myeloid cells, and in resting T-cells, respectively [4,12,13,14]. SAMHD1 also in vitro blocked infection by multiple other retroviruses such as HIV-2, FIV, bovine immunodeficiency virus (BIV), equine infectious anemia virus (EIAV), N-tropic murine leukemia virus (N-MLV), and B-tropic murine leukemia virus (B-MLV) [15]. Hence, it is expected that feline SAMHD1 will affect the innate immune response to FIV and FIV replication in a similar manner to the proposed mechanism of human SAMHD1. SAMHD1 is present in almost all tissues [10], and also prevents hepatitis B virus (HBV) replication in primary hepatocytes [16]. Flaviviruses have a (+)ssRNA genome, and replicate directly in the host cell cytoplasm. While SAMHD1 did not inhibit Chikungunya and Zika viruses in human fibroblasts, it suppressed IRF7 phosphorylation and inhibited NF-kB activation after infection, thereby indirectly augmenting anti-viral defense [17,18].

It has been proposed that SAMHD1 phosphorylation may also destabilize the tetrameric form of the molecule and, hence, act as a degradation signal during the normal cell cycle [19,20]. Interestingly, only the unphosphorylated or total SAMHD1 has antiviral activity. Almost all cycling and non-cycling cells express SAMHD1, but the antiviral activity is limited to non-cycling cells [21,22,23]. Full-length SAMHD1 had nuclease activity, which suggests that SAMHD1 might bind and degrade ssRNA, ssDNA, and RNA in DNA-RNA hybrids [24]. Binding to nucleic acids was identified in several studies [4,15,25], but recent findings showed that only ssRNA, including in vitro derived HIV-1 transcripts, was sensitive to the nuclease activity of SAMHD1 [26].

The full mechanism whereby SAMHD1 modulates innate and adaptive immune responses is incompletely characterized. Cyclic guanosine monophosphate (cGMP)–adenosine monophosphate (AMP) synthase (cGAS) is a cytoplasmic DNA sensor that detects double-stranded DNA, irrespective of the sequence [27]. Cytoplasmic dsDNA triggers the cGAS-STING-IRF3 pathway and subsequent production of type I IFN and other cytokines [28,29]. This pathway activates responses against DNA and RNA viruses [28]. However, cGAS does not bind to ssDNA; therefore, ssDNA viruses induce limited type I IFN [27,30]. Nevertheless, it was recently shown the stem-loop structure of the HIV-1 ssDNA transcript is highly immunostimulatory and does activate the cGAS-STING pathway [31]. SAMHD1 impairs reverse transcription [11,32], and thus lowers the level of viral cDNA. This low level of viral cDNA may preclude the trigger of this cytoplasmic DNA sensor, which in turn has been postulated as a mechanism of enabling HIV-1 to infect DCs without activating an effective antiviral response [32].

The gene encoding SAMHD1 was initially identified in a human dendritic cell cDNA library as an analogue to the mouse interferon (IFN)-induced gene MG11 [15,33]. The effect of IFNs on SAMHD1 regulation in human myeloid and T cells is unclear. Despite a transient increase in mRNA, type I IFNs did not enhance SAMHD1 protein expression in human primary dendritic cells, macrophages, or CD4^+^ T cells, but type I IFNs induced SAMHD1 expression in cell lines such as HEK 293T, HeLa, and U87 malignant glioma [34,35].

We previously reported that SAMHD1 is expressed in almost all feline tissues, and is most highly expressed at sites of potential feline immunodeficiency virus (FIV) entry and replication, including skin and mucosal epithelium, hemolymphatic, and spermatogenic tissues [36]. Anti-viral activity of SAMHD1 has been studied extensively in primate lentiviruses, specifically HIV-1. Similarity in viral structure, life cycle, and latency suggests SAMHD1 activity is likely to also regulate the host response to other lentiviruses.

Activation of target lymphocytes by cytokines such as IFNs could potentially enhance viral replication through increased receptor expression and dNTP availability. Hence, we hypothesized that SAMHD1 is upregulated by IFN exposure and moderates FIV replication. Specifically, in this study, we evaluated the effect of type I and II IFNs on SAMHD1 gene expression, transcription, cellular localization, and post-translational phosphorylation in feline immortalized and primary CD4^+^ T cells.

## 2. Materials and Methods

### 2.1. Cell Culture

The feline CD4^+^ cell line FeTJ was a kind gift of J. Yamamoto (University of Florida, Gainesville, FL, USA), and primary lymphocytes were isolated from the blood of cats euthanized for reasons unrelated to this study, in accordance with guidelines of the Canadian Council on Animal Care. Both primary lymphocytes and FeTJ cells were maintained in RPMI media supplemented with 10% fetal calf serum (FCS), 1% penicillin/streptomycin, and 50 µM of 2-mercaptoethanol (all from Invitrogen, Burlington, ON, Canada) in a humidified 5% CO_2_ incubator at 37 °C. Purified CD4^+^ lymphocytes were supplemented with 100 U/mL of human recombinant IL2 (Cell Sciences, Newburyport, MA, USA). Blood samples were obtained from three healthy FIV-negative cats. For gradient isolation of PBMC, whole blood was diluted with warm PBS, and then carefully layered over Ficoll with a density of 1.077 ± 0.001 g/mL at a ratio for blood:PBS:Ficoll of 1:1:1 (Ficoll Paque Plus, GE Healthcare, Mississauga, ON, Canada). After 30 min of centrifugation at 600× *g* without break, PBMC were collected and washed twice with PBS. The viable cells were counted using Trypan blue exclusion and re-suspended at 10^6^ cells/mL in a cell separation buffer (PBS, pH 7.2, 0.5% bovine serum albumin, 2 mM EDTA). Blood CD4^+^ lymphocytes were isolated from PBMC by immunomagnetic positive selection (Miltenyi Biotec, Auburn, CA, USA). Specifically, PBMC (10^7^ cells in 0.5 mL of cell separation buffer) were incubated with 1 μg of unlabelled anti-feline CD4 antibody (clone 3-4F4, Southern Biotech, Birmingham, AL, USA) for 10 min at 4 °C. Cells were washed twice and then incubated with 20 μL of goat anti-mouse IgG microbeads (Miltenyi Biotec) for 15 min at 4 °C. Cells were pelleted, washed twice, and then re-suspended in the cell separation buffer before being applied to a MACS-MS column following the manufacturer’s instructions. The positively selected cells were then cultured in complete RPMI media supplemented with 100 U/mL of recombinant human IL2. Cell purity and viability were determined by flow cytometry with antibodies against CD4 (clone 3–4F4), CD8 (clone fCD8, Southern Biotech), CD21 (clone LB21, Bio-Rad, Mississauga, ON, Canada), and 7AAD, respectively.

To determine whether IFNγ or IFNα affects SAMHD1 mRNA expression, FeTJ cells were treated with different concentrations of cytokine for 24 h, and samples were obtained before and then 6, 12, 18, and 24 h after treatment. To determine the optimal concentration of IFNγ, cells were treated with the midpoint concentration of ED_50_ of recombinant feline IFNγ, as recommended by the manufacturer (R&D systems, Minneapolis, MN, USA), plus two concentrations above and below (0.1, 0.2, 0.4, 0.8, and 1.2 ng/mL). In order to identify an optimal dose of IFNα, FeTJ cells were treated with 50, 300, 600, 1000, or 1200 U/mL of feline IFNα (PBL Assay Science, Piscataway, NJ, USA), similar to those that were used for T helper cell assays [37]. A concentration of 1000 U/mL induced a maximal increase in SAMHD1 mRNA, and was used in subsequent experiments.

### 2.2. RNA Extraction and Real-Time Quantitative PCR

Total RNA was isolated from cells before and after treatment using a Qiagen (Toronto, ON, Canada) RNeasy kit according to the manufacturer’s protocol. Double-stranded cDNA was synthesized from 1 μg of RNA using the QuantiTect Reverse Transcription Kit (Qiagen). Quantitative PCR (qPCR) was performed using a LightCycler 480 instrument (Roche Life Science, Laval, QC, Canada). The reaction mixture consisted of 10 μL SYBR Green Master Mix (Roche, Mississauga, ON, Canada), 0.5 μL of forward and reverse primer (concentration 10 μM), and 2 μL of cDNA in a final volume of 20 μL of PCR grade water. Amplification cycles were 10 min initial denaturation at 95 °C, followed by 45 cycles of denaturation at 95 °C for 20 s, annealing at 58 °C for 30 s, extension at 72 °C for 20 s, and then the final melting curve analysis. Six housekeeping gene candidates including β-actin, ribosomal protein S7 (RPS7), glyceraldehyde 3-phosphate dehydrogenase (GAPDH), ribosomal protein S19 (RPS19), and β-glucoronidase were evaluated. Beta-actin and RPS7 were selected as reference genes based on the production of a single melting curve peak, consistency of the standard curve, and a similar level of expression to the target gene, SAMHD1. SAMHD1 mRNA transcripts were normalized to the average of both reference genes using LightCycler 480 software, version 1.5.1.62.

Primer sequences were: SAMHD1 sense, 5′-CTT CCC TCA CCC TTT TAG CC-3′, and reverse 5′-CAG GAG GTA AAG AAC GAG CG-3′ [36]; β-actin sense 5′-CTC TTC CAG CCT TCC TTC CT-3′, and reverse 5′-ACT CCT GCT TGC TGA TCC AC-3′ [38]; and RPS7 sense 5′-GTC CCA GAA GCC GCA CTT TGA C-3′, and reverse 5′-CTC TTG CCC ACA ATC TCG CTC G-3′ [39].

### 2.3. Flow Cytometry

Cells (6 × 10^5^) were washed with PBS and then fixed and permeabilized with a Cytofix/Cytoperm Fixation/Permeabilization kit, according to manufacturer instructions (BD, Mississauga, ON, Canada). Cells were washed and re-suspended in 100 μL of perm-wash buffer (BD). The SAMHD1 antibody (clone OTI1A1, OriGene, Rockville, MD, USA) conjugated to Alexa Fluor 488, or the pSAMHD1 antibody (clone T592p, EMD Millipore, Billerica, MA, USA) that conjugated to Pacific Blue (Zenon labeling kit, Molecular Probes, Thermo Fisher, Mississauga, ON, Canada) was added, and the cells were incubated for 30 min at room temperature (RT) in the dark. SAMHD1 is highly conserved between species, and feline SAMHD1 has 77% amino acid identity with human SMAHD1. Motifs and predicted phosphorylation sites are conserved, and the specificity of antibody OT1A1 was previously assessed by Western blotting and immunohistochemistry using tissue microarrays (TMA) that used appropriate positive (human origin) and negative controls [36]. Clone T592p recognizes 11 amino acids around phosphorylated threonine at position 542 of human SAMHD1. This region is identical in feline and human SAMHD1. Negative controls consisted of cells treated identically and incubated with a non-binding antibody to human CXCR4 (clone 44747, R&D Systems) conjugated to the same fluorochromes. The cells were washed, pelleted, and re-suspended in 400 μL of flow cytometry (FC) buffer (PBS containing 5 mM EDTA, 2 mM NaN_3_, and 1% horse serum). Primary feline lymphocytes were analyzed first by incubation with the antibody to CD4 or CD8 (clones 3-4F4 and FT2, respectively; Southern Biotechnology, Birmingham, AL, USA), or CD21 (clone CA2.1D6, Bio-Rad, Mississauga, ON, Canada), all conjugated to PE, and subsequent permeabilization and staining for SAMHD1 occurred, as indicated above. All samples (minimum of 10000 events) were acquired with a FACSCanto II (BD) cytometer, and data were analyzed with FlowJo software (BD).

For nuclear staining, 2 × 10^6^ cells were washed in PBS, pelleted, and re-suspended in 100 μL of 0.2% Nonidet-P40 (Sigma-Aldrich, Oakville, ON, Canada) detergent for 2 min at room temperature to dissolve the cell membrane. After washing twice, nuclei were fixed, permeabilized, and stained, as described above.

Relative fluorescence intensity (RFI) was calculated by dividing the median fluorescence intensity (MFI) of cells analyzed with the SAMHD1 antibody by the MFI of cells prepared with the isotypic control antibody against CXCR4, both conjugated to Alexa 488. Relative fluorescence intensity for pSAMHD1 was calculated in the same manner for antibodies conjugated to Pacific Blue.

### 2.4. Western Blotting

Cytoplasmic and nuclear fractions were prepared using a commercial cell fractionation kit (NE-PER, Thermo Scientific) according to the manufacturer’s direction. After extraction, protein concentration in each sample was determined by spectrophotometry (Nanodrop 2000, Thermo Scientific). An equal amount of protein from each cytoplasmic or nuclear fraction was applied to wells for SDS-PAGE, followed by western blotting, as previously described [36]. Briefly, electrophoresis was performed in a 12% SDS-polyacrylamide gel under reducing conditions, followed by protein transfer to polyvinylidene fluoride (PVDF) membranes using a Trans-blot Turbo system (Bio-Rad). After blocking with 3% skim milk, the membranes were probed with the SAMHD1 antibody diluted into 10 mL of 1% *w*/*v* skim milk at 4 °C overnight. The following day, an anti-HDAC1 antibody (clone OTI5F9 OriGene) at a 1:1000 dilution as a nuclear protein loading control (Abcam, Brantford, CT, USA) or an anti-α-tubulin antibody (clone TU-01, Abcam) at a 1:300 dilution as a cytoplasmic protein loading control (Abcam) was applied for 2 h at room temperature. After washing and then incubation with horseradish peroxidase-conjugated secondary antibody (goat anti-mouse, 1:2000) for 30 min at room temperature, bands were visualized with a chemiluminescence detection kit (Clarity Western ECL substrate; Bio-Rad). Images were obtained with a Bio-Rad ChemiDoc gel documentation system and analyzed with Image Lab 6.0.0 software. A commercial lysate of HEK293T cells with transiently overexpressed SAMHD1 was used as a positive control (LY414526; OriGene).

### 2.5. Statistical Analysis

Each experiment was repeated independently at least thrice, and results are presented as mean ± SD. Statistical analysis was performed using SigmaStat 4.0 (Systat Software Inc., San Jose, CA, USA), using two-sided Student’s *t*-tests and Tukey’s multiple comparisons one-way ANOVA (GraphPad Software, La Jolla, CA, USA). Statistical significance was set at *p* < 0.05. Data were graphed with GraphPad Prism 6.0.

## 3. Results

Expression of SAMHD1 in multiple feline tissues was previously reported, but the effect of IFNs on SAMHD1 in immune cells is unknown [36]. This study was designed to determine whether type I or II IFN upregulates SAMHD1 transcription and induces posttranslational phosphorylation.

### 3.1. IFNγ Treatment Increases SAMHD1 mRNA

**Figure 1:**
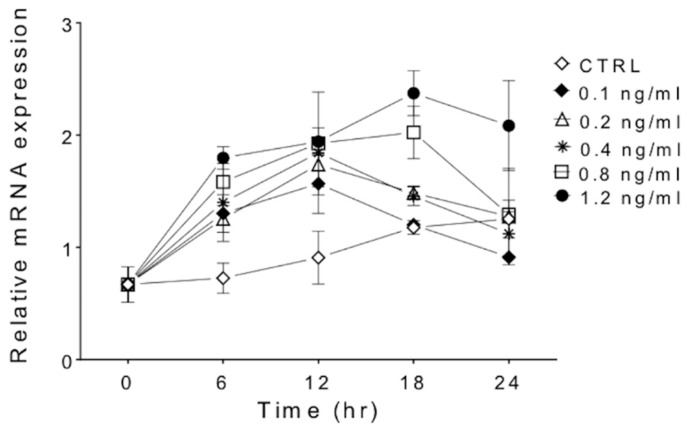
Quantification of sterile alpha motif and histidine aspartic domain-containing protein 1 (SAMHD1) mRNA by quantitative PCR shows a dose-dependent increase in response to increasing concentrations of IFNγ. Differences from the baseline were significant (*p* < 0.05) for all concentrations of IFNγ at 6 and 12 h, and for all concentrations of IFNγ except 0.1 ng/mL at 18 h. After 24 h, SAMHD1 mRNA was significantly higher than control only in cells treated with 1.2 ng/mL of IFNγ.

Expression of SAMHD1 mRNA was significantly (*p* < 0.05) higher in FeTJ cells at 6 and 12 h after treatment with 0.1, 0.2, 0.4, 0.8, and 1.2 ng/mL of IFNγ compared with controls (1.9–2.9 fold increase). At 18 h, cells exposed to 0.8 and 1.2 ng/mL of IFNγ had significantly higher SAMHD1 mRNA compared with that of the controls, and at 24 h, only cells treated with 1.2 ng/mL of IFNγ significantly differed from the controls (Figure 1). The induction of SAMHD1 mRNA was dose-dependent, with the highest concentration of IFNγ inducing the greatest increase in SAMHD1 mRNA expression. Specifically, 1.2 ng/mL of IFNγ resulted in SAMHD1 mRNA expression increases of 2.7, 2.9, 3.5, and 3.1-fold at 6, 12, 18, and 24 h post-treatment, respectively.

### 3.2. IFNγ Increases SAMHD1 Nuclear Translocation in FeTJ Cells

**Figure 2:**
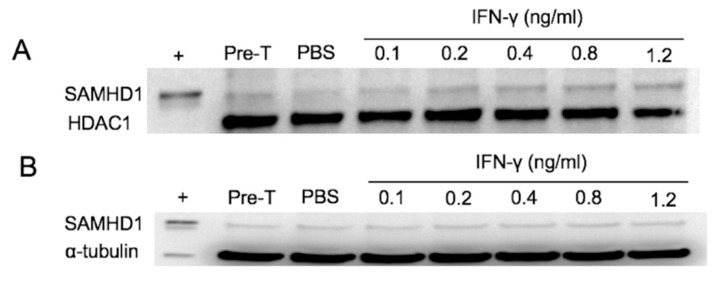
Detection of SAMHD1 by Western blotting in nuclear (**A**) and cytoplasmic (**B**) extracts of FeTJ cells treated with different concentrations of IFNγ for 6 h. Samples were collected pre-treatment and at 6 h post-treatment. HDAC1 and α-tubulin antibodies were used as nuclear and cytoplasmic protein loading controls, respectively.

Immunohistochemically, SAMHD1 is mainly localized to the nucleus, but immunoreactivity was also noted in the cytoplasm [36]. Transport of molecules into the nucleus depends on appropriate cell signals and the presence of a nuclear localization signal (NLS), and disruption of this motif results in accumulation of the molecule in the cytoplasm [40]. SAMHD1 has an NLS and is thought to function predominantly in the nucleus; therefore, the effect of IFNγ on cellular localization and translocation was investigated by Western blotting of nuclear and cytoplasmic fractions of FeTJ cells treated with IFNγ for 6 h. Increasing concentrations of IFNγ resulted in increased density of the nuclear SAMHD1 band, but changes in the density of cytoplasmic SAMHD1 were not appreciable (Figure 2A,B). Hence, nuclear translocation of SAMHD1 was sensitive to IFNγ in a dose-dependent manner.

For more precise quantification of SAMHD1, subsequent experiments used flow cytometric assessment of the phosphorylated and non-phosphorylated SAMHD1 protein in the whole cell and nuclear preparations of cells treated with 1.2 ng/mL of IFNγ.

### 3.3. IFNγ Treatment Increases Nuclear pSAMHD1 but Not Nuclear or Whole Cell SAMHD1

**Figure 3:**
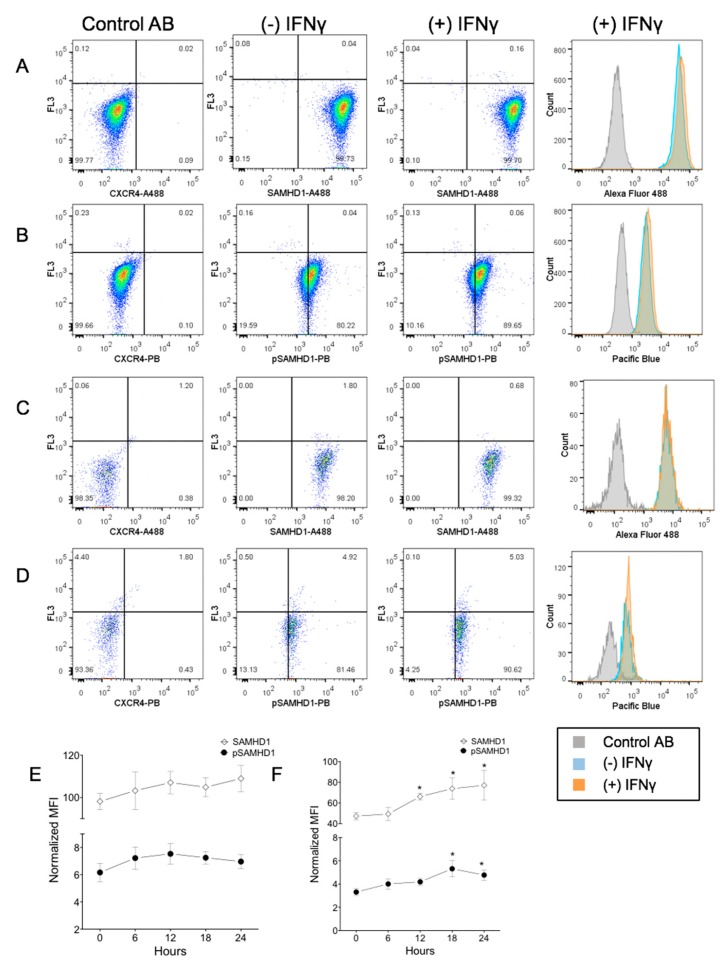
Flow cytometric assessment of a subcellular localization of total and phosphorylated SAMHD1. Whole cells (**A**,**B**) or nuclei (**C**,**D**) were stained with a fluorochrome-labeled non-binding CXCR4 antibody as the negative control, and with anti-SAMHD1 or anti-pSAMHD 6 h after treatment with 1.2 ng/mL of IFNγ. Both whole cells and nuclei were positive for SAMHD1 and pSAMHD1, but a significant increase in fluorescence associated with IFNγ treatment was noted only for the nuclear pSAMHD1 (**D**). Analysis over time showed that neither whole cell (**E**) SAMHD1 or pSAMHD1 increased significantly, but that nuclear (**F**) SAMHD1 and pSAMHD1 were significantly higher in treated cells at 12, 18 and 24 h, and at 18 and 24 h, respectively (*).

During 24 h of culture, aliquots of FeTJ cells were collected at 6 h intervals for flow cytometry. Detection of both SAMHD1 and pSAMHD1 varied minimally (<10%) in saline-treated cells, indicating stable expression during culture. At 18 h after treatment with 1.2 ng/mL of IFNγ, nuclear pSAMHD1 was significantly increased. However, at that timepoint, neither nuclear pSAMHD1 nor whole cell SAMHD1 or pSAMHD1 were significantly increased (Figure 3A–F). Analyzing results over time showed that nuclear SAMHD1 (Figure 3F) was significantly increased at 12, 18, and 24 h, and nuclear pSAMHD1 was significantly increased at 18 and 24 h. These findings were consistent with those of Western blotting.

### 3.4. IFNα Treatment Does Not Significantly Change SAMHD1 Phosphorylation or Localization

**Figure 4:**
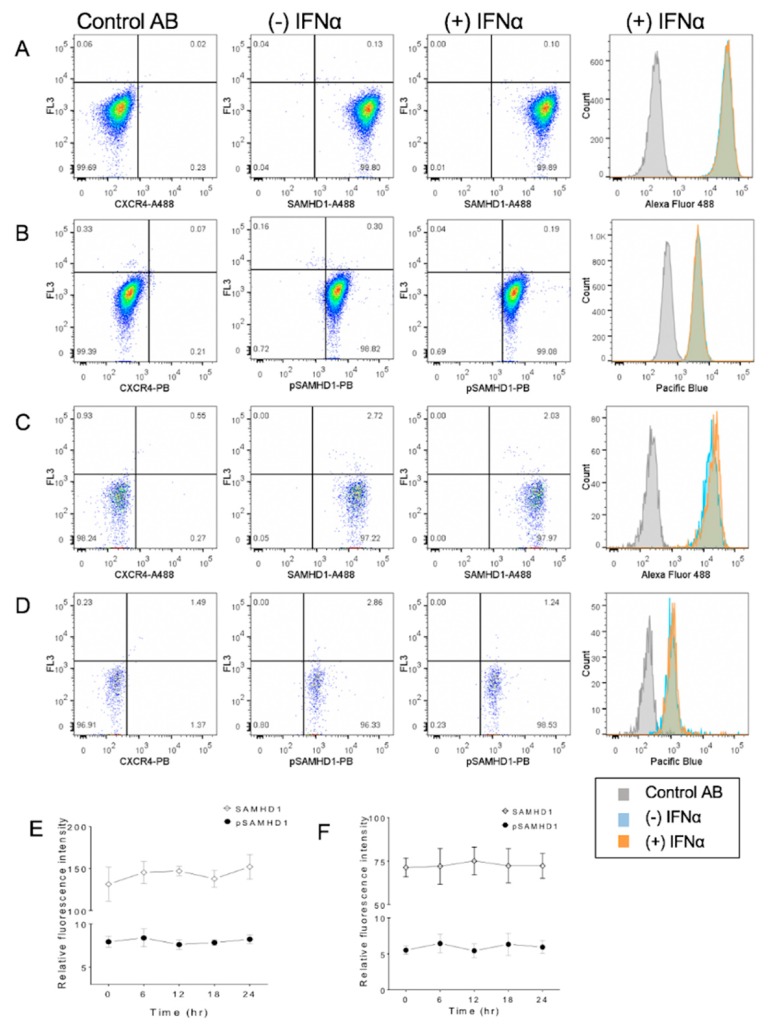
Flow cytometric assessment of subcellular localization of total and phosphorylated SAMHD1 in FeTJ cells treated with 1000 U/mL of IFNα. Samples were prepared as described above, and assessed after 6 h of treatment with IFNα (**A**–**D**). Treatment with IFNα did not induce a significant change in SAMHD1 or pSAMHD1 in either whole cell (**E**) or nuclear (**F**) preparations.

Flow cytometric analysis revealed that treatment of cells for 6 h with 1000 U/mL of IFNα did not significantly change SAMHD1 localization or phosphorylation in either whole cells or nuclei (Figure 4). Similarly, assessment over time did not reveal significant changes.

### 3.5. Primary Lymphocytes Contain Abundant SAMHD1 Protein That Is Not Further Increased by IFNγ Treatment

**Figure 5:**
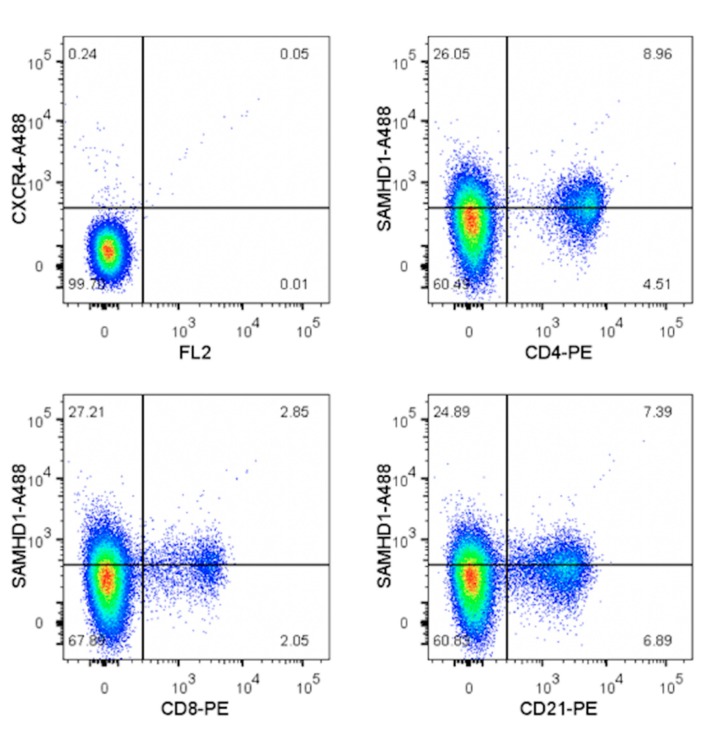
Detection of SAMHD1 relative to lymphocyte antigens on primary feline PBMC. Representative plots show staining for SAMHD1, CD4, CD8, and CD21. A proportion of both T and B lymphocytes are positive for SAMHD1.

Concurrent detection of SAMHD1 with CD4, CD8, or CD21 showed that among whole PBMC, both the primary T- and B-lymphocytes were highly positive for SAMHD1, and that expression was highest (~65%) in CD4^+^ lymphocytes (Figure 5).

**Figure 6:**
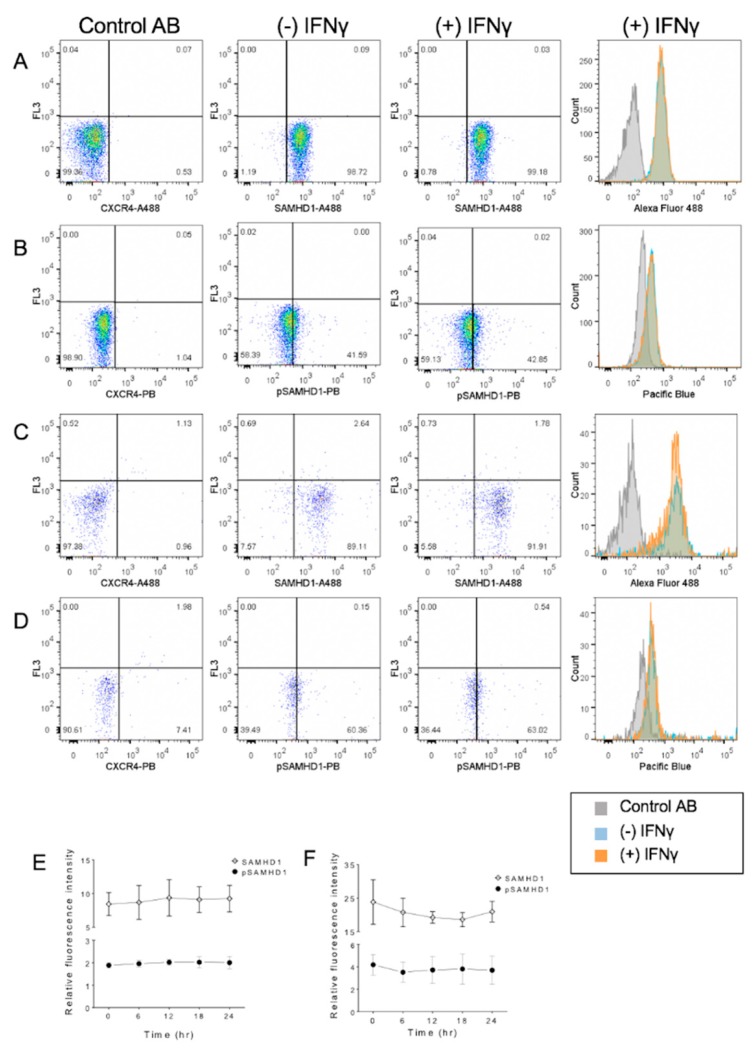
Flow cytometric detection of cellular localization of total and phosphorylated SAMHD1 in purified primary feline CD4^+^ lymphocytes maintained in IL-2. Samples consisted of lymphocytes stained with a fluorochrome-labeled non-binding CXCR4 antibody as the negative control, and whole cells (**A**,**B**) or nuclei (**C**,**D**) were stained with anti-SAMHD1 or anti-pSAMHD 6 h after treatment with IFNγ. No significant differences in relative fluorescence attributable to IFNγ treatment were observed. Summary of results over time in whole cells (**E**) and nuclei (**F**).

Once purified, CD4^+^ lymphocytes were maintained with IL2, and after 48 h of culture, they were uniformly positive for SAMHD1 (Figure 6A). Treatment with IFNγ did not significantly increase detection of either SAMHD1 or pSAMDh1 in the whole cells or nuclei.

## 4. Discussion

SAMHD1 was first identified in mouse macrophages as an IFNγ inducible protein [41]. As a component of dNTP degrading enzymes, SAMHD1 plays a crucial role in the regulation and balance of the cellular dNTP pool [2,5]. SAMHD1 is most highly expressed during cellular quiescence (G0 phase) and minimally expressed during replication (S phase) [42]. SAMHD1 hydrolyzes dNTP’s, and thereby, prevents accumulation of excess dNTPs in non-dividing quiescent cells [43]. Through this finely-tuned activity, SAMHD1 modulates anti-proliferative and tumor-suppressive functions, and the life cycle of several DNA and retroviruses [1,14,16].

The activity of SAMHD1 is dGTP-regulated, and dGTP is a substrate and also an activator of SAMHD1 [4]. Upon combined activation by both GTP and other dNTPs, SAMHD1 molecules form a tetramer, which has biological activity [6,7]. This active tetrameric form remains functional for hours, even after the dNTP concentration decreases [44]. Functionally, inactivating mutations in SAMHD1 can cause in an imbalance in the dNTP pool, which in turn could contribute to improper nucleotide insertion, mutations, and genomic instability [1,2]. On the other hand, a high concentration of cytosolic nucleotides can trigger innate immune responses [45]. Mutations in SAMHD1 have been associated with a broad spectrum of diseases: the Catalogue of Somatic Mutations In Cancer (COSMIC) currently lists 195 *SAMHD1* mutations found in various cancers, including breast, central nervous system, hematopoietic, lymphoid, kidney, intestine, skin, thyroid, and others [46].

SAMHD1, as a dNTP triphosphohydrolase enzyme, is conserved from bacteria to humans [44]. SAMHD1 contains the two major structural SAM and HD domains. The SAM domain can potentially interact with other domains, including SAM domains of other proteins and also nucleic acids. The regulatory functions of human and mouse SAM domains differ. In humans, the SAM domain is not involved in nuclease activity, and the HD domain is sufficient for dNTP depletion and, therefore, antiviral activity. However, the SAM domain is necessary for allosteric activation and dNTP hydrolysis in mouse SAMHD1 [4,15,47]. The feline SAMHD1 (fSAMHD1) sequence (GenBank Id. 2012037) is most highly similar to that of humans [36], and in vitro human SAMHD1 in addition to HIV-1 also blocked FIV, HIV-2, BIV, EIAV, N-MLV, and B-MLV [15].

IFNs are the main cytokines used to initiate cell-intrinsic antiviral defenses [48]. As the first line of antiviral defense, IFNs induce interferon-stimulated genes (ISG) that encode antiviral proteins [49]. Examples of ISG products that have retroviral restricting activity are APOBEC3G, TRIM5, tetherin, SAMHD1, and MOV10 [50]. Recent studies assessing the influence of IFNs on the production and activity of restriction factors have expanded our knowledge about some functions of the innate immune system. For example, IFNα induces APOBEC3 in macrophages [51] and TRIM5α in a number of primate cell lines, including HeLa cells (a human cervical cell cancer line), Vero cells (African green monkey kidney epithelial cells), CMMT cells (a rhesus macaque mammary tumor cell line), and owl monkey kidney cells [52]. From several studies evaluating the effect of IFNs on SAMHD1 expression, it was concluded that SAMHD1 is IFN-inducible in a cell type dependent manner. Despite a transient increase in mRNA, type I IFN did not increase SAMHD1 protein expression in primary human dendritic cells, macrophages, or CD4^+^ T cells, but IFN induced SAMHD1 expression in cell lines such as HEK 293T, HeLa, and U87 malignant glioma [34,35].

In this study we showed that in FeTJ cells, SAMHD1 mRNA increased in response to IFNγ. IFNγ is a type II IFN produced mainly by lymphocytes, while IFNα is a type I IFN produced by monocytes and non-leukocytes [53,54]. FeTJ cells are IL2 independent, CD4^+^, immortalized cells that are susceptible to FIV infection [36]. Increased expression of SAMHD1 after treatment with IFNγ might impart greater resistance to FIV replication. SAMHD1 restricting activity is also regulated by post-translational modification [2,5,6]. Specifically, it has been proposed that phosphorylation could destabilize the tetrameric form of SAMHD1 and act as a degradation signal [19,20]. Phosphorylation of SAMHD1 at threonine 592 by the A2 cyclin-dependent kinase (CDK) 1 occurs prior to the cell cycle S phase, and concurrent with an increase in intracellular dNTPs [6,21,23]. Soon after this discovery, SAMHD1 was recognized as a retroviral restriction factor that reduced cellular dNTPs below a threshold critical for viral reverse transcription [55]. Other potential antiviral mechanisms are binding and subsequent exonuclease activity against ssRNA and DNA [24,56]. It had been suggested that SAMHD1’s putative exonuclease activity might be due to experimental conditions resulting in contamination, since other researchers failed to detect nuclease activity at the SAMHD1 active site [57]. However, Ryoo et al. (2016) suggested that SAMHD1 is a phosphorolytic rather than a hydrolytic ribonuclease. Phosphorolytic degradation of RNA produces nucleoside diphosphates, whereas hydrolytic degradation of RNA generates nucleoside monophosphates [26].

Here, the treatment of FeTJ cells with IFNγ increased both phosphorylated and non-phosphorylated nuclear SAMHD1 protein concentration in a dose-dependent manner. This suggests that active SAMHD1 might affect lentiviral replication in the nucleus by different mechanisms of activity, but also that phosphorylation might modulate activity. The effect of phosphorylation on the activity of SAMHD1 is not fully understood [5]. Phosphorylation by CDKs at T592 negatively regulates the restricting function and RNAse activity of SAMHD1 [21,58]. One of the complex viral strategies to evade SAMHD1 restriction is phosphorylation of T592 by beta- and gamma- herpes viruses. These viruses have a conserved protein kinase called CHPK, which mimics cellular CDK1 and CDK2 activity to phosphorylate cell-cycle-related proteins [59]. Hence, it remains uncertain whether an increase in the phosphorylated form of SAMHD1 after IFNγ exposure confers the resistance of cells to retrovirus infection or replication.

It was postulated that IFNα treatment would induce phosphatase activity to dephosphorylate SAMHD1, as described by Cribier et al. (2013) in activated CD4^+^ T cells, monocyte-derived macrophages (MDM), monocyte-derived dendritic cells (MDDC), and THP-1 cells [21]. A similar effect was not noted in astrocytes or microglial cells [10,60]. Our results indicate that IFNα treatment affected neither phosphorylated nor total SAMHD1 in FeTJ cells. Since a dose previously shown to activate human Th1 cells and feline PBMC was used, it is likely that SAMHD1 is minimally responsive to IFNα stimulation [61,62]. Regulation of SAMHD1 expression and activity is incompletely defined, and recently it was proposed that micro-RNA 181a (miR-181a) might reduce SAMHD1 expression by mRNA degradation [63]. The miR-181 is an important target of many cytokines, and IFNs increased SAMHD1 expression through the downregulation of miR-181a and miR-30a in human monocytes [64].

The strengths of our study were optimization and application of a flow cytometric assay to detect both the whole cell and nuclear total, as well as the phosphorylated SAHMD1. Flow cytometry is a highly sensitive and quantitative technique for enumerating molecules in a large number of cells or particles, and it is more precise than semi-quantitative assessments by Western blotting [65]. Moreover, since mRNA stability may be highly variable and the quantification of protein is more biologically relevant, the flow cytometric assay was deemed both more sensitive and specific. Previous studies assessed only cell extracts, which could obscure intracellular locations of proteins such as SAMHD1. The limitations of this study were that the effect of different amounts of SAMHD1 and pSAMHD1 in nuclei and whole cells was not assessed for their effect on FIV replication.

All primary feline lymphocytes expressed a relatively high level of total SAMHD1, with the highest expression in CD4^+^ lymphocytes. The latter cells are the primary target of FIV, which fits with the ability of SAMHD1 to modulate dNTP concentrations and limit viral replication. These findings are similar to those in humans where naïve (CD45RA^+^, CCR7^+^), central memory (CD45RA^−^, CCR7^+^), and effector memory (CD45RA^−^, CCR7^−^) CD4^+^ lymphocytes are the primary target of HIV infection [66]. Assays to delineate feline CD4^+^ subpopulations are not established, but it is likely that akin to HIV, FIV targets specific resting lymphocyte populations.

Interleukin (IL) 2 supplementation is required for in vitro survival of primary lymphocytes, and it affects T-helper, T-regulatory, and activated T-cells [67,68]. Since both γc-cytokines and SAMHD1 are involved in cell cycle regulation, their interaction might affect cell cycling and also SAMHD1’s antiviral activity [69]. IL2 did not affect SAMHD1 protein expression in phytohemagglutinin activated CD4^+^ cells [11], but induced phosphorylation of SAMHD1 in primary CD4^+^ lymphocytes [69]. In our studies, after exposure to IL2, nearly all CD4^+^ T-cells became positive for SAMHD1. Phosphorylated SAMHD1 was not as highly expressed in primary CD4^+^ lymphocytes as in FeTJ cells. Overall, this might indicate that the active, non-phosphorylated form of SAMHD1 predominates in primary cells, regardless of IFNγ exposure. IFNs could have other mechanisms to enhance SAMHD1 activity against retroviruses.

Findings from this study are in agreement with the widespread, though cell-type dependent, expression of SAMHD1 [36]. Exposure to IFNγ but not IFNα increased SAMHD1 mRNA expression, and nuclear SAMHD1 and pSAMHD1 protein expression, in a feline T-lymphocyte cell line. Primary lymphocytes had high SAMHD1 after IL2 stimulation, which did not further increase with the addition of IFNγ. A more detailed understanding of SAMHD1 function with and without post-translational modifications is needed to ascribe definitive roles in viral restriction and cell cycle regulation.

## References

[1] Mauney C.H., Hollis T. (2018). SAMHD1: Recurring roles in cell cycle, viral restriction, cancer, and innate immunity. Autoimmunity.

[2] Pai C.C., Kearsey S.E. (2017). A Critical Balance: dNTPs and the Maintenance of Genome Stability. Genes (Basel).

[3] Pajalunga D., Franzolin E., Stevanoni M., Zribi S., Passaro N., Gurtner A., Donsante S., Loffredo D., Losanno L., Bianchi V. (2017). A defective dNTP pool hinders DNA replication in cell cycle-reactivated terminally differentiated muscle cells. Cell Death Differ..

[4] Goldstone D.C., Ennis-Adeniran V., Hedden J.J., Groom H.C., Rice G.I., Christodoulou E., Walker P.A., Kelly G., Haire L.F., Yap M.W. (2011). HIV-1 restriction factor SAMHD1 is a deoxynucleoside triphosphate triphosphohydrolase. Nature.

[5] Tramentozzi E., Ferraro P., Hossain M., Stillman B., Bianchi V., Pontarin G. (2018). The dNTP triphosphohydrolase activity of SAMHD1 persists during S-phase when the enzyme is phosphorylated at T592. Cell Cycle.

[6] Sze A., Olagnier D., Lin R., van Grevenynghe J., Hiscott J. (2013). SAMHD1 host restriction factor: a link with innate immune sensing of retrovirus infection. J. Mol. Biol..

[7] Zhu C., Gao W., Zhao K., Qin X., Zhang Y., Peng X., Zhang L., Dong Y., Zhang W., Li P. (2013). Structural insight into dGTP-dependent activation of tetrameric SAMHD1 deoxynucleoside triphosphate triphosphohydrolase. Nat. Commun..

[8] Baldauf H.-M., Stegmann L., Schwarz S.-M., Ambiel I., Trotard M., Martin M., Burggraf M., Lenzi G.M., Lejk H., Pan X. (2017). Vpx overcomes a SAMHD1-independent block to HIV reverse transcription that is specific to resting CD4 T cells. Proc. Natl. Acad. Sci. USA.

[9] Hofmann H., Logue E.C., Bloch N., Daddacha W., Polsky S.B., Schultz M.L., Kim B., Landau N.R. (2012). The Vpx lentiviral accessory protein targets SAMHD1 for degradation in the nucleus. J. Virol..

[10] Schmidt S., Schenkova K., Adam T., Erikson E., Lehmann-Koch J., Sertel S., Verhasselt B., Fackler O.T., Lasitschka F., Keppler O.T. (2015). SAMHD1’s protein expression profile in humans. J. Leukoc. Biol..

[11] Baldauf H.M., Pan X., Erikson E., Schmidt S., Daddacha W., Burggraf M., Schenkova K., Ambiel I., Wabnitz G., Gramberg T. (2012). SAMHD1 restricts HIV-1 infection in resting CD4(+) T cells. Nat. Med..

[12] Hollenbaugh J.A., Gee P., Baker J., Daly M.B., Amie S.M., Tate J., Kasai N., Kanemura Y., Kim D.-H., Ward B.M. (2013). Host factor SAMHD1 restricts DNA viruses in non-dividing myeloid cells. PLoS Pathog..

[13] Kim E.T., Roche K.L., Kulej K., Spruce L.A., Seeholzer S.H., Coen D.M., Diaz-Griffero F., Murphy E.A., Weitzman M.D. (2019). SAMHD1 Modulates Early Steps during Human Cytomegalovirus Infection by Limiting NF-κB Activation. Cell Rep..

[14] Kim E.T., White T.E., Brandariz-Núñez A., Diaz-Griffero F., Weitzman M.D. (2013). SAMHD1 restricts herpes simplex virus 1 in macrophages by limiting DNA replication. J. Virol..

[15] White T.E., Brandariz-Nunez A., Valle-Casuso J.C., Amie S., Nguyen L., Kim B., Brojatsch J., Diaz-Griffero F. (2013). Contribution of SAM and HD domains to retroviral restriction mediated by human SAMHD1. Virology.

[16] Sommer A.F., Rivière L., Qu B., Schott K., Riess M., Ni Y., Shepard C., Schnellbächer E., Finkernagel M., Himmelsbach K. (2016). Restrictive influence of SAMHD1 on Hepatitis B Virus life cycle. Sci. Rep..

[17] Daep C.A., Muñoz-Jordán J.L., Eugenin E.A. (2014). Flaviviruses, an expanding threat in public health: focus on dengue, West Nile, and Japanese encephalitis virus. J. Neurovirol..

[18] Wichit S., Hamel R., Zanzoni A., Diop F., Cribier A., Talignani L., Diack A., Ferraris P., Liegeois F., Urbach S. (2019). SAMHD1 Enhances Chikungunya and Zika Virus Replication in Human Skin Fibroblasts. Int. J. Mol. Sci..

[19] Gelais C.S., Kim S.H., Maksimova V.V., Buzovetsky O., Knecht K.M., Shepard C., Kim B., Xiong Y., Wu L. (2018). A cyclin-binding motif in human SAMHD1 is required for its HIV-1 restriction, dNTPase activity, tetramer formation, and efficient phosphorylation. J. Virol..

[20] St Gelais C., Kim S.H., Ding L., Yount J.S., Ivanov D., Spearman P., Wu L. (2016). A Putative Cyclin-binding Motif in Human SAMHD1 Contributes to Protein Phosphorylation, Localization, and Stability. J. Biol. Chem..

[21] Cribier A., Descours B., Valadão A.L.C., Laguette N., Benkirane M. (2013). Phosphorylation of SAMHD1 by cyclin A2/CDK1 regulates its restriction activity toward HIV-1. Cell Rep..

[22] White T.E., Brandariz-Nuñez A., Martinez-Lopez A., Knowlton C., Lenzi G., Kim B., Ivanov D., Diaz-Griffero F. (2017). A SAMHD1 mutation associated with Aicardi-Goutières Syndrome uncouples the ability of SAMHD1 to restrict HIV-1 from its ability to downmodulate type I interferon in humans. Hum. Mutat..

[23] White T.E., Brandariz-Nunez A., Valle-Casuso J.C., Amie S., Nguyen L.A., Kim B., Tuzova M., Diaz-Griffero F. (2013). The retroviral restriction ability of SAMHD1, but not its deoxynucleotide triphosphohydrolase activity, is regulated by phosphorylation. Cell Host Microbe.

[24] Beloglazova N., Flick R., Tchigvintsev A., Brown G., Popovic A., Nocek B., Yakunin A.F. (2013). Nuclease activity of the human SAMHD1 protein implicated in the Aicardi-Goutieres syndrome and HIV-1 restriction. J. Biol. Chem..

[25] Goncalves A., Karayel E., Rice G.I., Bennett K.L., Crow Y.J., Superti-Furga G., Burckstummer T. (2012). SAMHD1 is a nucleic-acid binding protein that is mislocalized due to aicardi-goutieres syndrome-associated mutations. Hum. Mutat..

[26] Ryoo J., Hwang S.-Y., Choi J., Oh C., Ahn K. (2016). SAMHD1, the Aicardi-Goutieres syndrome gene and retroviral restriction factor, is a phosphorolytic ribonuclease rather than a hydrolytic ribonuclease. Biochem. Biophys. Res. Commun..

[27] Li T., Chen Z.J. (2018). The cGAS–cGAMP–STING pathway connects DNA damage to inflammation, senescence, and cancer. J. Exp. Med..

[28] Ma Z., Damania B. (2016). The cGAS-STING Defense Pathway and Its Counteraction by Viruses. Cell Host Microbe.

[29] Coquel F., Silva M.-J., Técher H., Zadorozhny K., Sharma S., Nieminuszczy J., Mettling C., Dardillac E., Barthe A., Schmitz A.-L. (2018). SAMHD1 acts at stalled replication forks to prevent interferon induction. Nature.

[30] Ishii K.J., Coban C., Kato H., Takahashi K., Torii Y., Takeshita F., Ludwig H., Sutter G., Suzuki K., Hemmi H. (2006). Erratum: A Toll-like receptor–independent antiviral response induced by double-stranded B-form DNA. Nat. Immunol..

[31] Herzner A.-M., Hagmann C.A., Goldeck M., Wolter S., Kübler K., Wittmann S., Gramberg T., Andreeva L., Hopfner K.-P., Mertens C. (2015). Sequence-specific activation of the DNA sensor cGAS by Y-form DNA structures as found in primary HIV-1 cDNA. Nat. Immunol..

[32] Chen S., Bonifati S., Qin Z., Gelais C.S., Wu L. (2019). SAMHD1 suppression of antiviral immune responses. Trends Microbiol..

[33] De Silva S., Hoy H., Hake T.S., Wong H.K., Porcu P., Wu L. (2013). Promoter methylation regulates SAMHD1 gene expression in human CD4+ T cells. J. Biol. Chem..

[34] Goujon C., Schaller T., Galao R.P., Amie S.M., Kim B., Olivieri K., Neil S.J., Malim M.H. (2013). Evidence for IFNalpha-induced, SAMHD1-independent inhibitors of early HIV-1 infection. Retrovirology.

[35] St Gelais C., de Silva S., Amie S.M., Coleman C.M., Hoy H., Hollenbaugh J.A., Kim B., Wu L. (2012). SAMHD1 restricts HIV-1 infection in dendritic cells (DCs) by dNTP depletion, but its expression in DCs and primary CD4+ T-lymphocytes cannot be upregulated by interferons. Retrovirology.

[36] Asadian P., Finnie G., Bienzle D. (2018). The expression profile of sterile alpha motif and histidine-aspartate domain-containing protein 1 (SAMHD1) in feline tissues. Vet. Immunol. Immunopathol..

[37] Golding A., Rosen A., Petri M., Akhter E., Andrade F. (2010). Interferon-alpha regulates the dynamic balance between human activated regulatory and effector T cells: implications for antiviral and autoimmune responses. Immunology.

[38] Achleitner A., Clark M.E., Bienzle D. (2011). T-regulatory cells infected with feline immunodeficiency virus up-regulate programmed death-1 (PD-1). Vet. Immunol. Immunopathol..

[39] Penning L.C., Vrieling H.E., Brinkhof B., Riemers F.M., Rothuizen J., Rutteman G.R., Hazewinkel H.A. (2007). A validation of 10 feline reference genes for gene expression measurements in snap-frozen tissues. Vet. Immunol. Immunopathol..

[40] Schaller T., Pollpeter D., Apolonia L., Goujon C., Malim M.H. (2014). Nuclear import of SAMHD1 is mediated by a classical karyopherin α/β1 dependent pathway and confers sensitivity to Vpx MAC induced ubiquitination and proteasomal degradation. Retrovirology.

[41] Lafuse W.P., Brown D., Castle L., Zwilling B.S. (1995). Cloning and characterization of a novel cDNA that is IFN-γ-induced in mouse peritoneal macrophages and encodes a putative GTP-binding protein. J. Leukoc. Biol..

[42] Franzolin E., Salata C., Bianchi V., Rampazzo C. (2015). The Deoxynucleoside Triphosphate Triphosphohydrolase Activity of SAMHD1 Protein Contributes to the Mitochondrial DNA Depletion Associated with Genetic Deficiency of Deoxyguanosine Kinase. J. Biol. Chem..

[43] Lahouassa H., Daddacha W., Hofmann H., Ayinde D., Logue E.C., Dragin L., Bloch N., Maudet C., Bertrand M., Gramberg T. (2012). SAMHD1 restricts the replication of human immunodeficiency virus type 1 by depleting the intracellular pool of deoxynucleoside triphosphates. Nat. Immunol..

[44] Hansen E.C., Seamon K.J., Cravens S.L., Stivers J.T. (2014). GTP activator and dNTP substrates of HIV-1 restriction factor SAMHD1 generate a long-lived activated state. Proc. Natl. Acad. Sci. USA.

[45] Crow Y.J., Manel N. (2015). Aicardi–Goutières syndrome and the type I interferonopathies. Nat. Rev. Immunol..

[46] The Catalogue of Somatic Mutations in Cancer (COSMIC). https://cancer.sanger.ac.uk/cosmic/gene/analysis?ln=SAMHD1.

[47] Buzovetsky O., Tang C., Knecht K.M., Antonucci J.M., Wu L., Ji X., Xiong Y. (2018). The SAM domain of mouse SAMHD1 is critical for its activation and regulation. Nat. Commun..

[48] Schoggins J.W. (2018). Recent advances in antiviral interferon-stimulated gene biology. F1000Research.

[49] Doyle T., Goujon C., Malim M.H. (2015). HIV-1 and interferons: who’s interfering with whom?. Nat. Rev. Microbiol..

[50] Zheng Y.H., Jeang K.T., Tokunaga K. (2012). Host restriction factors in retroviral infection: promises in virus-host interaction. Retrovirology.

[51] Peng G., Lei K.J., Jin W., Greenwell-Wild T., Wahl S.M. (2006). Induction of APOBEC3 family proteins, a defensive maneuver underlying interferon-induced anti–HIV-1 activity. J. Exp. Med..

[52] Carthagena L., Parise M.C., Ringeard M., Chelbi-Alix M.K., Hazan U., Nisole S. (2008). Implication of TRIMalpha and TRIMCyp in interferon-induced anti-retroviral restriction activities. Retrovirology.

[53] Ferreira V.L., Borba H.H.L., Bonetti A.D.F., Leonart L., Pontarolo R. Cytokines and Interferons: Types and Functions. https://www.intechopen.com/books/autoantibodies-and-cytokines/cytokines-and-interferons-types-and-functions.

[54] Lee A.J., Ashkar A.A. (2018). The dual nature of type I and type II interferons. Front. Immunol.

[55] Welbourn S., Dutta S.M., Semmes O.J., Strebel K. (2013). Restriction of virus infection but not catalytic dNTPase activity are regulated by phosphorylation of SAMHD1. J. Virol..

[56] Tüngler V., Staroske W., Kind B., Dobrick M., Kretschmer S., Schmidt F., Krug C., Lorenz M., Chara O., Schwille P. (2013). Single-stranded nucleic acids promote SAMHD1 complex formation. J. Mol. Med..

[57] Seamon K.J., Sun Z., Shlyakhtenko L.S., Lyubchenko Y.L., Stivers J.T. (2015). SAMHD1 is a single-stranded nucleic acid binding protein with no active site-associated nuclease activity. Nucleic Acids Res..

[58] Ryoo J., Choi J., Oh C., Kim S., Seo M., Kim S.Y., Seo D., Kim J., White T.E., Brandariz-Nunez A. (2014). The ribonuclease activity of SAMHD1 is required for HIV-1 restriction. Nat. Med..

[59] Zhang K., Lv D.-W., Li R. (2019). Conserved Herpesvirus Protein Kinases Target SAMHD1 to Facilitate Virus Replication. Cell Rep..

[60] Jin C., Peng X., Liu F., Cheng L., Xie T., Lu X., Wu H., Wu N. (2016). Interferon-induced SAMHD1 expression in astrocytes and microglia is mediated by miR-181a. AIDS.

[61] Jarry A., Malard F., Bou-Hanna C., Meurette G., Mohty M., Mosnier J.-F., Laboisse C.L., Bossard C. (2017). Interferon-alpha promotes Th1 response and epithelial apoptosis via inflammasome activation in human intestinal mucosa. Cellular Mol. Gastroenterol. Hepatol..

[62] Morrison J.H., Guevara R.B., Marcano A.C., Saenz D.T., Fadel H.J., Rogstad D.K., Poeschla E.M. (2014). Feline immunodeficiency virus envelope glycoproteins antagonize tetherin through a distinctive mechanism that requires virion incorporation. J. Virol..

[63] Jin C., Peng X., Liu F., Cheng L., Lu X., Yao H., Wu H., Wu N. (2014). MicroRNA-181 expression regulates specific post-transcriptional level of SAMHD1 expression in vitro. Biochem. Biophys. Res. Commun..

[64] Riess M., Fuchs N.V., Idica A., Hamdorf M., Flory E., Pedersen I.M., König R. (2017). Interferons induce expression of SAMHD1 in monocytes through down-regulation of miR-181a and miR-30a. J. Biol. Chem..

[65] Hoss F., Rolfes V., Davanso M.R., Braga T.T., Franklin B.S., De Nardo D., De Nardo C. (2018). Detection of ASC speck formation by flow cytometry and chemical cross-linking. Innate Immune Activation. Methods in Molecular Biology.

[66] Descours B., Cribier A., Chable-Bessia C., Ayinde D., Rice G., Crow Y., Yatim A., Schwartz O., Laguette N., Benkirane M. (2012). SAMHD1 restricts HIV-1 reverse transcription in quiescent CD4(+) T-cells. Retrovirology.

[67] Overwijk W.W., Schluns K.S. (2009). Functions of γC cytokines in immune homeostasis: Current and potential clinical applications. Clin. Immunol..

[68] Read K.A., Powell M.D., McDonald P.W., Oestreich K.J. (2016). IL-2, IL-7, and IL-15: multistage regulators of CD4+ T helper cell differentiation. Exp. Hematol..

[69] Coiras M., Bermejo M., Descours B., Mateos E., García-Pérez J., López-Huertas M.-R., Lederman M.M., Benkirane M., Alcamí J. (2016). IL-7 Induces SAMHD1 Phosphorylation in CD4+ T Lymphocytes, Improving Early Steps of HIV-1 Life Cycle. Cell Rep..

